# Anatomical relation between S1 sacroiliac screws’ entrance points and superior gluteal artery

**DOI:** 10.1186/s13018-018-0713-5

**Published:** 2018-01-18

**Authors:** Yong Zhao, Libo You, Wei Lian, Dexin Zou, Shengjie Dong, Tao Sun, Shudong Zhang, Dan Wang, Jingning Li, Wenliang Li, Yuchi Zhao

**Affiliations:** 1 Orthopaedics Department, Yantai Shan Hospital, 91#, Jiefang Road, Yantai, 264008 Shandong Province People’s Republic of China; 2 Operating Room, Yantai Shan Hospital, 91#, Jiefang Road, Yantai, 264008 Shandong Province People’s Republic of China; 3 CT/MR Department, Yantai Shan Hospital, 91#, Jiefang Road, Yantai, 264008 Shandong Province People’s Republic of China

**Keywords:** Superior gluteal artery, Sacroiliac screw, Pelvis, Anatomy, CTA

## Abstract

**Background:**

To conduct radiologic anatomical study on the relation between S1 sacroiliac screws’ entry points and the route of the pelvic outer superior gluteal artery branches with the aim to provide the anatomical basis and technical reference for the avoidance of damage to the superior gluteal artery during the horizontal sacroiliac screw placement.

**Methods:**

Superior gluteal artery CTA (CT angiography) vascular imaging of 74 healthy adults (37 women and 37 men) was done with 128-slice spiral CT (computed tomography). The CT attendant-measuring software was used to portray the “safe bony entrance area” (hereinafter referred to as “Safe Area”) of the S1 segment in the standard lateral pelvic view of three-dimensional reconstruction. The anatomical relation between S1 sacroiliac screws’ Safe Area and the pelvic outer superior gluteal artery branches was observed and recorded. The number of cases in which artery branches intersected the Safe Area was counted. The cases in which superior gluteal artery branches disjointed from the Safe Area were identified, and the shortest distance between the Safe Area and the superior gluteal artery branch closest to the Safe Area was measured.

**Results:**

Three cases out of the 74 sample cases were excluded from this study as they were found to have no bony space for horizontal screw placement in S1 segment. Among the remaining 71 sample cases, there are 32 cases (45.1%) where the deep superior branch of superior gluteal artery passes through the Safe Area of S1 entrance point. There was no distinguishing feature and rule on how the deep superior branches and the Safe Area overlapped. In the 39 cases in which superior gluteal artery branches disjointed from the Safe Area, the deep superior branches of superior gluteal artery were the branches closest to the Safe Area and the part of the branch closest to the Safe Area was located in front of the widest part of the Safe Area. The shortest distance between the deep superior branch and the Safe Area is 0.86 ± 0.84 cm.

**Conclusion:**

There is a high risk of accidental injury of the deep superior branches of superior gluteal artery in the process of S1 sacroiliac screw placement. Even if the entry points are located in the safe bony entrance area, the absolute secure placement cannot be assured. We suggest that great attention should be paid to make thorough preoperative plans.

## Background

Sacroiliac screw placement has almost become the golden rule for treating posterior pelvic ring injuries [1], but there are still certain risks of injury to superior gluteal artery in the process of screw placement, thus resulting in hemorrhagic shock, pseudoaneurysm, and other serious complications [2, 3]. Although there are limited reports on iatrogenic injury to superior gluteal artery, however as it may trigger large amount of bleeding and is tough to treat within limited short time, it raises the hidden risk of medical disputes and thus its serious consequence makes it worthy of high attention. In response to the concerns raised above, this article shows a radiological anatomical study regarding the relation between the direction of the superior gluteal artery and its branches in the outer part of the sacrum and the entrance points of S1 sacroiliac screws so as to provide anatomical basis and technical reference for the avoidance of injuries to artery and its relevant serious complications in the process of clinical placement.

## Methods

The ethics committee of Yantai Shan Hospital approved this study. Written informed consents from all participants involved in this study were obtained.

### Material

The study cohort is comprised of 74 pelvis CTA (CT angiography) cases selected from enhanced abdominal CT database of our hospital randomly, including 37 men and 37 women, whose age varied from 20 to 86 years old (56 years old on average). In the case selection process, whichever pelves found of tumor, fracture, or variation were excluded from the research scope.

### Equipment and scan mode

Philips Ingenuity 128-slice spiral CT was used to scan the inspected lying supinely from head to foot. The scanning parameter was 3-mm collimation (reduced to 1 mm), 0.625-mm interval, 120 kV, and 200–300 mA. The pitch was 1.375. Sixty to 80 ml (370 mgI/ml) ultravist was injected through the elbow vein, and the rates were 3–3.5 ml/s. The scanning will start automatically by monitoring the contrast agent concentration of the aorta. The nebula workstation was used to analyze the data. MPR and 3D reconstruction were performed in all the cases.

### Measure methods and contents

**Fig. 1:**
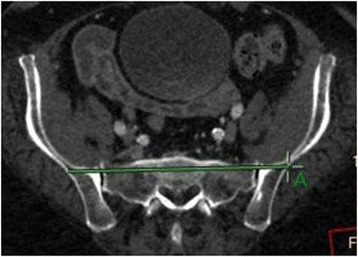
Two horizontal lines were drawn through the bilateral sacroiliac joints from the right to the left in S1 segment, and they were limited in the bone substance and could not break both the front and back margins of sacrum, canales sacralis, nor sacral foramen. The two lines were defined respectively as passing through the anterior and posterior edges of the bone substance of the pelvic posterior ring. The intersection points of the two lines and the outer margins of ilium were marked and named as point As (S1 sacroiliac screw entrance points)

**Fig. 2:**
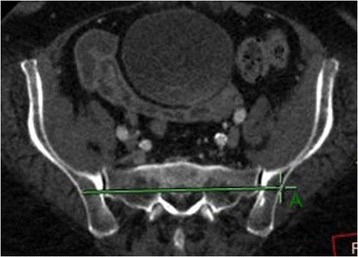
Two horizontal lines were drawn through the bilateral sacroiliac joints from the right to the left in S1 segment, and they were limited in the bone substance and could not break both the front and back margins of sacrum, canales sacralis, nor sacral foramen. The two lines were defined respectively as passing through the anterior and posterior edges of the bone substance of the pelvic posterior ring. The intersection points of the two lines and the outer margins of ilium were marked and named as point As (S1 sacroiliac screw entrance points)

**Fig. 3:**
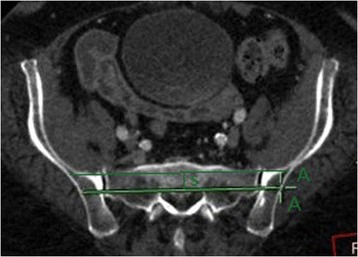
The length between the two horizontal lines aforesaid in Figs. 1 and 2 (the width of safe insertion region) was named as S

At first, the scan direction was unified to parallel the superior margin of S1 and the horizontally scanned image of S1 segment was observed. Two horizontal lines were drawn through the bilateral sacroiliac joints from the right to the left and within the bone substance without breaking both the front and back margins of sacrum, canales sacralis, nor sacral foramen. The two lines were defined respectively as passing through the anterior and posterior edges of the bone substance of the pelvic posterior ring. The intersection points of the two lines and the outer margins of ilium were marked and named as point As (S1 sacroiliac screw entrance points) (Figs. 1 and 2). The length between the two lines (the width of safe insertion region) was named as S (Fig. 3). Several point As were marked at five layers, i.e., the upper layer when the two lines first appeared, the middle layer where S is the longest, the bottom layer where the two lines disappear, the layer in between the upper and middle layers, and the layer in between the middle and bottom layers.

**Fig. 4:**
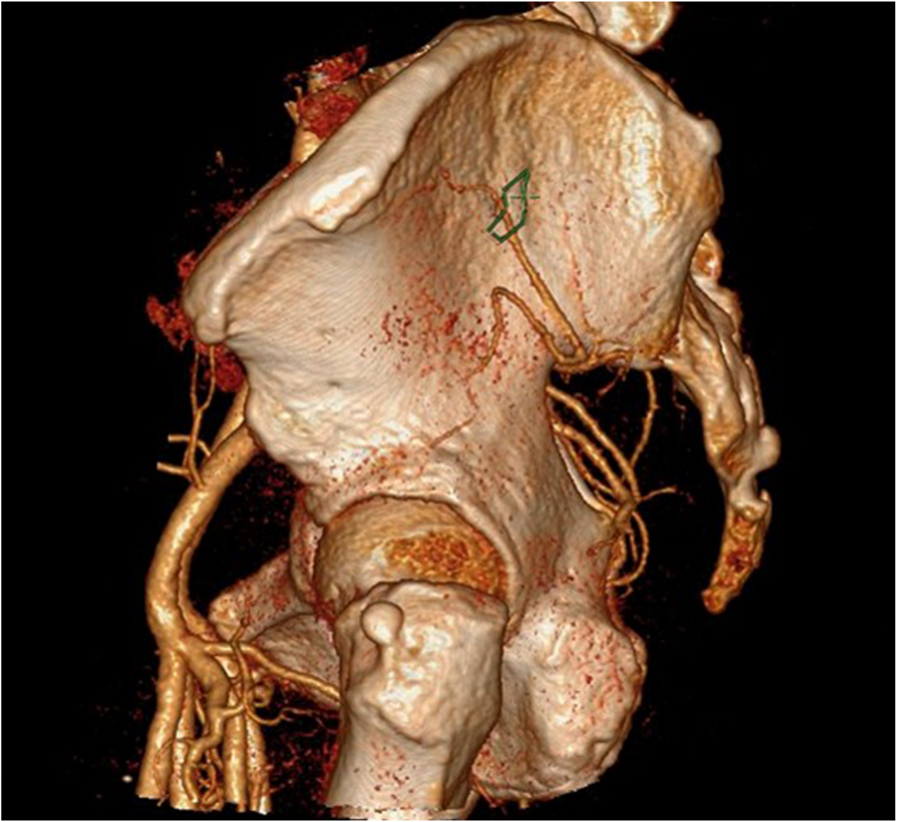
The point As were connected to form a region (*green borders* in the pictures) named as the S1 sacroiliac screw’s safe entrance bony area (Safe Area for short). The relations of the Safe Area and various branches of superior gluteal artery outside of the pelvis were observed. In Fig. 4, the deep superior branch of superior gluteal artery just passes through the Safe Area. In contrast, the branches of superior gluteal artery and the Safe Area do not intersect in Fig. 5, “*D*” means the shortest distance between the bony Safe Area and the deep superior branch

**Fig. 5:**
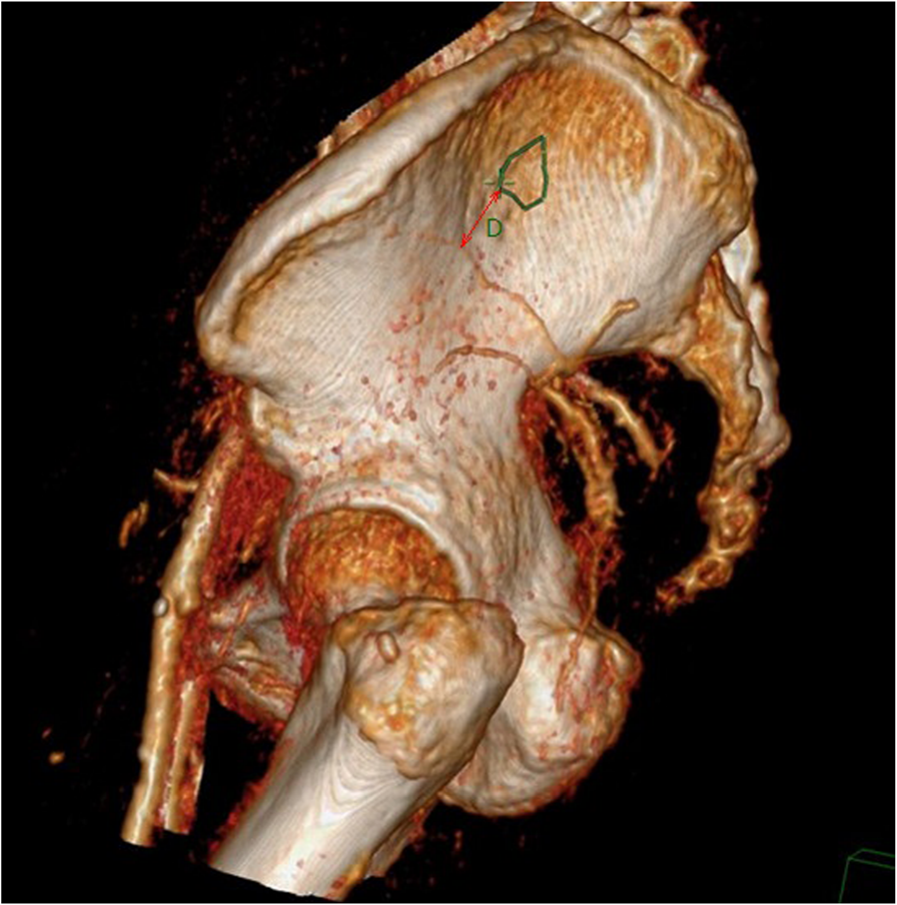
The point As were connected to form a region (*green borders* in the pictures) named as the S1 sacroiliac screw’s safe entrance bony area (Safe Area for short). The relations of the Safe Area and various branches of superior gluteal artery outside of the pelvis were observed. In Fig. 4, the deep superior branch of superior gluteal artery just passes through the Safe Area. In contrast, the branches of the superior gluteal artery and the Safe Area do not intersect in Fig. 5, “*D*” means the shortest distance between the bony Safe Area and the deep superior branch

Secondly, the point As (the S1 sacroiliac screw entrance points) were observed and measured on the CT reconstruction images. The positions of pelves were adjusted to make the top edge of pubic symphysis and the bottom edge of sacrum at the same horizontal, and the pelvic CT reconstruction images were adjusted to the standard lateral positions. The 10-point As were connected to form a region named as the S1 sacroiliac screw’s safe entrance bony area (hereinafter referred to as Safe Area). The relations of the Safe Area and various branches of superior gluteal artery outside of the pelvis were observed. The cases in which superior gluteal artery passes through the Safe Area were recorded. Likely, the cases where the superior gluteal artery and the safe entrance area do not intersect were also recorded, and the shortest distance (D) between the branches of superior gluteal artery and the Safe Area was measured (Figs. 4 and 5).

### Statistics

The statistical description was done with SPSS 13.0. The distribution characteristic of the data was described, and the concentrating and dispersing trends of the data were described.

## Results

Among the 74 sample cases, 3 cases were excluded from this study as they were found to have no bony space for horizontal screw placement in S1 segment. Among the remaining 71 sample cases, there are 32 cases (45.1%) where the deep superior branch of superior gluteal artery passes through the Safe Area of S1 entrance point. After our observation on such overlapping sample cases, we did not find any obvious features and rules on how they overlapped. Our study showed that, in the 39 sample cases where the superior gluteal artery and the entrance point do not intersect, the deep superior branch of superior gluteal artery is the artery branch closest to the Safe Area and the part closest to the Safe Area is located in front of the widest part of the Safe Area. Since all the *D* data measured was in line with normal distribution, it is denoted via mean and standard deviation $\left(\overline{X}\pm S\right)$. The shortest distance (*D*_S_) between the bony Safe Area and the deep superior branch was 0.86 ± 0.84 cm.

## Discussion

During the placement process, sacroiliac screws are likely to puncture the bony structure, thus causing injury to iliac blood vessels, presacral venous plexus, L5 S1 nerve root, and sacral nerves. In order to avoid such iatrogenic injuries, doctors must have overall and complete command of pelvic morphometry supplemented with clear image scanning during the surgery. That is why the previous studies [4] were mainly focused on the above issues. However, those studies were based on the anatomical relations between the screw and the bony structure of pelvic posterior ring. In other words, they studied from the screw’s entry into the outer plate of iliac bone while there were rare relevant studies on the process of the screw’s reaching to the bone through the skin.

Superior gluteal artery is an important branch of the internal iliac artery, passing across the pelvis. Any pulling violence or direct violence may cause injury to it, individually or coexisting with pelvic fractures. There were various reported cases abroad on the iatrogenic injuries in this regard [2, 3, 5, 6], mainly concentrated on the iliac crest bone graft surgery, the posterior superior iliac acetabular revision surgery, and percutaneous sacroiliac screw placement. Once the superior gluteal artery is injured, due to fast bleeding and massive hemorrhage, it may easily lead to serious complications such as hemorrhagic shock. As the blood vessels are deep and the broken ends of vascular retracts, it is impossible to do direct vision ligation but instead to do packing hemostasis immediately and to perform the internal iliac artery embolization, which would prolong the surgery and hospital stay, increase the economic burden on patients and the risk of pseudoaneurysm, and thus trigger the risk of medical disputes. As iliac crest bone graft surgery and the posterior superior iliac acetabular revision surgery are open operations, the surgeon has a chance to view the superior gluteal artery branches, so theoretically the risk of superior gluteal artery injury is relatively low; on the contrary, the risk of iatrogenic injury to superior gluteal artery during the percutaneous sacroiliac screw placement is much higher. Therefore, the Safe Area of sacroiliac screw entrance point would be truly safe only when it also takes into account the anatomical features of the superior gluteal artery in the outside area of the pelvis.

The superior gluteal artery usually springs from the internal iliac artery and is the largest branch derived backward from the latter, directing to the backward and downward within the pelvis, along with the separation of nutritional branches and muscular branches to supply the iliac, external iliac muscle, piriformis, obturator muscle, etc., getting out of the pelvis at the lower edge of the greater sciatic notch and the upper edge of piriformis, and then immediately separating into the deep branch and the superficial branch. The foresaid superficial branch goes inward and upward and dominates the upper one third of the gluteus maximus and part of the skin behind the sacrum, while the deep branch generates numerous perforating branches to supply the gluteal medius and the gluteus minimus and goes among them, dividing into the deep superior branch and the deep inferior branch. The deep superior branch continues along with the direction of blood vessel trunk from the upper edge of the gluteus minimus until the anterior superior spine, while the deep inferior branch goes diagonally through the gluteus minimus to the femoral great trochanter.

The entrance point of sacroiliac screw locates between the rear of the large sciatic notch and the iliac wing [7, 8], and theoretically speaking, it has the chance to overlap with superior gluteal artery branches located outside the pelvis or with its lateral view of the body surface projection. In general, there are two sacroiliac screw placement directions. One is directed diagonally from the outer back into the front inner applicable to the sacroiliac joint dislocation; the other is directed horizontally from the outer to the inner applicable to longitudinal fractures of the sacrum (tile C sacral fractures). The latter can also be applicable to sacroiliac joint dislocation. Although the Safe Area for placement for the latter is smaller than that of the former, we have been still working on the relevant radiological anatomy and biomechanics study on the Safe Area of placement in the latter direction due to its irreplaceability in treating the longitudinal fractures of the sacrum which bears lowest stability of posterior pelvic ring [9–11].

Our study showed that, S1 entrance points have large chance of overlapping with the deep superior branch of superior gluteal artery. As per our observation of the overlapping samples, we did not find any obvious features and rules on how they overlapped. In addition, our study showed that, in the sample cases where the superior gluteal artery and the entrance points do not intersect, the most risky area that the superior gluteal artery passes was exactly located in front of the widest part of the placement area, the shortest distance (*D*_S_) between the bony Safe Area and the deep superior branch being only 0.86 cm. The abovementioned observation results revealed that, even if the entrance point is located in the safe bony area, it is hard to secure the absolute safety of the placement. In our opinion, the guide pin/drill shall be inserted into the safe bony area accurately on one time because any seemingly minor positioning deviations may cause injury to the superior gluteal artery branch, especially in the wider zone of safe bony area which should be paid more attention.

The previous relevant studies on superior gluteal artery iatrogenic injury were individual case reports and focused on S1 sacroiliac screw. The case report of Marmor et al. [2] believed that, the abnormal growth of the superficial branch of the superior gluteal artery will lead to the increasing risk of relevant iatrogenic injury during the process of sacroiliac screw placement. Collinge et al. [12] believed that, the deep superior branch of the superior gluteal artery is the most vulnerable part in the process of sacroiliac screw placement. We believe that, the different injury records of different branches may be due to the different directions of screw placements. It was well documented that, in the experimental study of Collinge et al. [12], the direction of sacroiliac screw placement is set to be from outer lower back to inner upper front and it was observed that, the deep superior branch of superior gluteal neurovascular bundle has the most chance of being injured by the entrance point. Our study showed that, theoretically, S1 sacroiliac screw entrance point has the most chance of overlapping with the deep superior branch of the superior gluteal artery, although the entrance point of horizontal placement is slightly anterior. The reasons may be the following: first, it may be due to the wider scope of the sagittal plane space between the deep superior branch and the superficial branch of the superior gluteal artery; second, it could not be denied that the measuring error was increased because the superior gluteal artery and the superior gluteal nerves were combined to be superior gluteal neurovascular bundle in previous studies.

Altman et al. [5] reported individual cases on injuries to superior gluteal neurovascular bundle caused in the process of clinical sacroiliac screw placement, and it was reported that it triggered rapid bleeding at the entrance point when a 69-year-old patient was receiving the operation of percutaneous guide pin placement. They attributed such injury to aortic calcification viewable by CT. They speculated that such arterial injury may be due to direct injury or tractive injury by the guide pin. Marmor et al.’s case [2] showed that the superior gluteal artery injury may happen to young patients who do not have aortic calcification but have vascular malformations in superior gluteal artery. Maled et al. [3] reported cases where the fixation of two 7.3-mm sacroiliac screws in the S1 segment resulted in superior gluteal artery pseudoaneurysm. Stephen [6] reported one case where the superior gluteal artery pseudoaneurysm incurred 15 days after percutaneous sacroiliac screw placement, and he speculated that pseudoaneurysm may result from several factors and may be due to the original pelvis injury or the direct injury during the process of screw placement. It is thus evident that, although the characteristics of the superior gluteal artery and the expression of superior gluteal artery injuries may vary, all without exception have relevance to the direct violence of sacroiliac screw placement.

Results of our study showed that, even if we exclude the factor of vascular malformation (mutation), theoretically, there is a high chance that S1 sacroiliac screw entrance points and the superior gluteal artery may overlap, that is to say, the risk of accidental injury to superior gluteal artery in the process of S1 sacroiliac screw placement is still high. We believe that the superior gluteal artery in its nature is not a reason to iatrogenic injury. Accordingly, in clinical practice, we should fully anticipate the possibility of artery mutation and furthermore should also be cautious during the screw placement so as to reduce, to the maximum, the risk of injury to superior gluteal artery. On the one hand, we recommend that guide pin/screw protective sleeve should be designed and used, and that the guide pin and screws should be placed slowly in order to reduce the chance of injury to the superior gluteal artery that may be caused by the guide pin/screws. On the other hand, we suggest the guide pin (or drill) should be positioned successfully in one time. Since the entrance point Safe Area, comparatively speaking, could be accurately positioned via the C-arm fluoroscopy and computer navigation, accordingly the failure of one-time positioning should be attributed to the far distance between the skin and the iliac plate or the softness of the guide pin making it impossible to place the guide pin or drill into the designated area of iliac plate accurately as planned, which is the most common problem in the clinical sacroiliac screw placement. Therefore, we suggest the auxiliary stable guide device for sacroiliac screw placement should be designed as soon as possible to increase, to the maximum extent, the chance of successful one-time guide pin or drill positioning and thus to minimize the risk of iatrogenic superior gluteal artery injury.

## Conclusion

There is a high risk of accidental injury to the deep superior branches of superior gluteal artery in the process of S1 sacroiliac screw placement. Even if entry points are located in the safe bony entrance area, absolutely secured placement cannot be assured. We suggest that great attention must be paid to make thorough preoperative plans.

## Abbreviations

CT: Computed tomography
CTA: CT angiography
*D*_S_: The shortest distance between the bony Safe Area and the deep superior branch
